# Polyvinyl alcohol coating releasing fungal blastospores improves kill effect of attract-and-kill beads

**DOI:** 10.1186/s13568-023-01575-2

**Published:** 2023-07-11

**Authors:** Katharina M. Hermann, Alexander Grünberger, Anant V. Patel

**Affiliations:** 1 Faculty of Engineering and Mathematics, Fermentation and Formulation of Biologicals and Chemicals, Hochschule Bielefeld – University of Applied Sciences and Arts, Bielefeld, Germany; 2 Faculty of Technology, Multiscale Bioengineering, Bielefeld University, Bielefeld, Germany

**Keywords:** PVA, Coating, *Metarhizium brunneum*, Blastospores, Attract-and-kill, Formulation

## Abstract

Polyvinyl alcohol (PVA) is a biodegradable, water-soluble polymer with excellent film forming properties, commonly studied or used as tablet coating, food packaging or controlled release fertilizers. Attract-and-kill (AK) beads are sustainable, microbial alternatives to synthetic soil insecticides, whose onset of lethal effect largely depend on how fast the encapsulated entomopathogenic fungus forms virulent conidia. Therefore, the objective of this study was to develop a water-soluble coating accelerating the kill effect of AK beads by immediately releasing virulent *Metarhizium brunneum* CB15-III blastospores. We assessed three PVA types (PVA 4-88, 8-88, 10-98) which differed in their degree of hydrolysis or molecular weight for their ability to release viable blastospores from thin films after drying at 60–40 °C, and examined how polyethylene glycol and soy-lecithin impact the blastospore survival. Finally, we evaluated the effectiveness of coated AK beads in a bioassay against *Tenebrio molitor* larvae. The blastospore release rate quadrupled within the first 5 min with decreasing molecular weight and degree of hydrolysis, with PVA 4-88 releasing 79 ± 19% blastospores*.* Polyethylene glycol and soy-lecithin significantly increased the blastospore survival to 18–28% for all three PVA types. Coated beads exhibited a uniform, 22.4 ± 7.3 µm thin coating layer, with embedded blastospores, as confirmed by scanning electron microscopy. The blastospore coating increased the mortality rate of *T. molitor* larvae over uncoated AK beads, decreasing the median lethal time from 10 to 6 days. Consequently, the blastospore coating accelerated the kill effect of regular AK beads. These findings pave the way to enhanced pest control efficacy from coated systems such as beads or seeds.

**Graphical Abstract:**

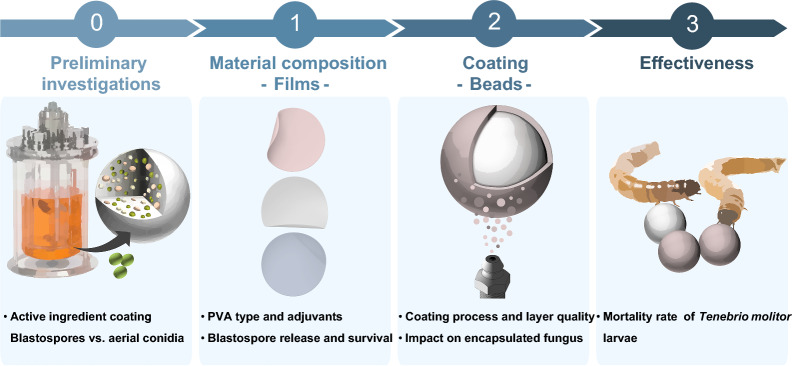

**Supplementary Information:**

The online version contains supplementary material available at 10.1186/s13568-023-01575-2.

## Key points

- PVA enables immobilization of virulent blastospores on alginate beads.
- Blastospore coating accelerates kill effect of attract-and-kill beads.
- PEG4000 and soy lecithin improve blastospore survival in PVA films.

**Supplementary Information:**

The online version contains supplementary material available at 10.1186/s13568-023-01575-2.

## Introduction

In 2050, with an estimated population of 9.7 billion people (Food and Agriculture Organization 2018), the total global food demand is expected to have increased by 35–56%, relative to 2010 (Van Dijk et al. 2021). Current agricultural practices, including excessive use of fertilizers and chemical pesticides, degrade the soils and agro-ecosystems health in pursuing sufficient food quantity and quality (Ayoub 1999; Wood and Goulson 2017; Tripathi et al. 2020). A major and widespread pest that is increasingly damaging a variety of crops is the click beetle larvae, known as wireworms (*Coleoptera: Elateridae*). Wireworms can be very destructive and cause severe economic losses to farmers (Parker and Howard 2001; Vernon and van Herk 2013; Poggi et al. 2018). Two chemical pesticides became the main class of insecticides routinely used for controlling wireworms. (i) Neonicotinoids (i.e. imidacloprid, clothianidin and thiamethoxam) were commonly used as prophylactic treatment to protect seeds and seedlings, and, (ii) phenyl pyrazoles (e.g. fipronil) were the most effective wireworm insecticides available because they rapidly killed wireworms of all species upon contact, and also had a latent toxicity, thus, killing wireworms even several months after exposure (Van Herk et al. 2008; Vernon et al. 2013; Morales-Rodriguez and Wanner 2015). With the abandonment of the aforementioned chemical insecticides due to environmental concerns, and the lack of alternatives, the control of wireworms has become more difficult. Consequently, the demand for conventional, or better yet, biological control options has increased tremendously (European Food Safety Authority 2013; Vernon and van Herk 2013; Poggi et al. 2018).

A highly promising biological approach is the attract-and-kill (AK) strategy in which pests are attracted by CO_2_ toward an entomopathogenic fungus (EPF) (Brandl et al. 2017). EPFs have been used worldwide as biocontrol agents in various formulation types and crops since the 1960s (Faria and Wraight 2007). Building on the fundamental work of Pereira and Roberts (1991), Humbert et al. (2017a; b) and Przyklenk et al. (2017) developed a calcium alginate/starch bead formulation, containing CO_2_ producing *Saccharomyces cerevisiae* and entomopathogenic *Metarhizium brunneum.* This formulation can achieve a long-lasting effect by forming masses of aerial conidia (hereafter referred to as conidia) on the surface of each bead*.* However, employing EPFs as the active ingredient still has drawbacks that need to be addressed. Currently, when *M. brunneum* or *Beauveria bassiana* are encapsulated in calcium alginate, conidia are formed more slowly than CO_2_ is produced (Vemmer et al. 2016; Hermann et al. 2021). This delayed sporulation creates a time lag between attraction and infection of the pests, and most likely reduces the efficacy of the mycoinsecticide. Consequently, the immediate supply of virulent spores, either conidia or blastospores, needs to be guaranteed. Conidia are more robust, whereas blastospores are considered more virulent and easier to propagate (Hall 1979; Miranpuri and Khachatourians 1990; Agosin et al. 1997; Wyatt et al. 2013; Wang et al. 2013; Jaronski 2014; Alkhaibari et al. 2016, 2017; Yun et al. 2017). Blastospores are thin-walled, yeast-like cells that originally form in the hemolymph of insects during infection (Hänel 1982), but can also be obtained in vitro by submerged cultivation. Blastospores can be considered a by-product of mycelium production, and vice versa.

Recent agricultural research engaging with biodegradable coatings, primarily focused on coating either fertilizers to control their release (Han et al. 2009; Nguyen et al. 2019; Wesołowska et al. 2021) or seeds to enhance plant growth, yield and health (Gorim and Asch 2012; Damasceno et al. 2013; Vercelheze et al. 2019; Ma 2019). In this context, film coating has become widely accepted as a reliable approach improving the productivity of crops. Film coating involves applying a thin layer, usually of synthetic or biological polymers mixed with plasticizers, solvents, pigments and beneficial microorganisms by means of a drum dryer or fluidized bed dryer (Ma 2019), and choosing the appropriate coating material is crucial.

Polyvinyl alcohol (PVA) is a biocompatible, biodegradable, synthetic polymer with excellent film forming ability. Moreover, it is water-soluble, nontoxic, noncarcinogenic and inexpensive (Wong and Parasrampuria 1996; DeMerlis and Schoneker 2003). Due to its versatile properties it is used and studied in many different applications, including wound dressing (Sung et al. 2010), drug delivery (Brough et al. 2016a; b), tablet coating (Koo et al. 2011), detergent packaging (Byrne et al. 2021), food packaging (Schiessl et al. 2022), seed coating (Vercelheze et al. 2019), fertilizer coating (Han et al. 2009), and permanent immobilization or encapsulation of cells (Wu and Wisecarver 1992; Lee and Cho 2010; Damasceno et al. 2013). However, to our knowledge, there is no study on targeted and fast spore release for pest control. PVA is a polyhydric alcohol containing secondary hydroxyl groups on alternate carbon atoms and is represented by the formula (C_2_H_4_O)_n_. Its structural monomer vinyl alcohol is thermodynamically unstable and cannot exist in the free form. Therefore, for commercial production, vinyl acetate is polymerized to polyvinyl acetate which is subsequently hydrolyzed to PVA. Various PVA grades exist, depending on the extent of hydrolysis and, consequently, unconverted fractions of polyvinyl acetate (Koltzenburg et al. 2017).

Polyethylene glycol (PEG) is a water-soluble, nontoxic and biodegradable polymer, with specific properties depending on its molecular weight. PEG has been used for decades in cosmetics, food, pharmaceutical formulations and plant protection products as adjuvant, e.g. plasticizer. Plasticizers can reduce the crystallinity and melting temperature of PVA and increase water absorption, water vapor transmission and biodegradability (Lim and Wan 1994, 1995; Ismail and Zaaba 2011; Shojaee Kang Sofla et al. 2020; Ucpinar Durmaz and Aytac 2021).

Soy lecithin (Lec) is a naturally derived, important component providing manifold functions in numerous industrial applications. It is widely used as lubricant and surfactant in food, pharmaceutical or agrochemical industry (Van Nieuwenhuyzen 1976; Scholfield 1981; Baseeth and Sebree 2010; List 2015).

Here, we hypothesize that one way to overcome the delayed conidia release by the encapsulated EPF in the AK base bead is through additional adhesion and immediate release of virulent spores. Hence, the objective of this study was to develop a water-soluble film coating, suitable for blastospore immobilization, to immediately release these virulent spores in soil (‘fast acting’), and therefore, bridge the time until the encapsulated EPF in the base bead forms virulent conidia (‘depot effect’). Consequently, we aimed to accelerate the kill effect of AK beads, without impairing the growth of the encapsulated EPF. As coating material, three PVA differing in their degree of hydrolysis and their molecular weight as well as PEG and Lec were investigated. The first part of this work evaluates the PVA films regarding their blastospore release rate and blastospore survival. The second part of the work examines the bead coating and whether the coating layer affects the growth of the encapsulated EPF. Finally, the third part of the work clarifies how the blastospore coating alters the lethality of AK beads and thus their effectiveness against *Tenebrio molitor* larvae.

## Methods

### Materials

All chemical compounds used in this study were acquired from Carl Roth GmbH (Karlsruhe, Germany) and concentrations are given as (w/w), unless stated otherwise.

### Microorganisms, culture conditions and preparation of biomass

*Saccharomyces cerevisiae* H205 was provided ready for use by Deutsche Hefewerke GmbH (Nürnberg, Germany). The product contains pure moist yeast biomass with a cell concentration of 1.02 ± 0.13 × 10^10^ cells/g.

*Metarhizium brunneum* strain CB15-III (DSM 33077, German Collection of Microorganisms and Cell Cultures, Braunschweig, Germany) was obtained from Prof. Dr. Stefan Vidal (Agricultural Entomology, Department for Crop Science, Georg-August-University Goettingen, Germany). Cultures were prepared as described previously by Hermann et al. (2021). Conidia, derived from freeze-dried biomass (mycelium and blastospores) and cultivated on Potato Dextrose Agar (PDA) at 25 °C for 7 days, were utilized for inoculation of submerged cultures. Pre-cultures were inoculated with a final concentration of 1 × 10^6^ conidia/mL and cultivated in 100 mL liquid medium modified according to Krell et al. (2018) (7.5% glucose, 4.0% ANiPept (ANiMOX GmbH, Berlin, Germany, batch No. 1176), 7.0% polyethylene glycol 200) at 23 °C and 150 rpm in 250 mL baffled shake flasks for 72 h. Main cultures were inoculated with 5 mL [i.e. 5.0% (v/v)] vegetative pre-culture containing blastospores and mycelium and were also cultivated for 72 h. The mycelial biomass which was later used for encapsulation, was harvested under aseptic conditions by vacuum filtration through paper dishes (Whatman No. 4, qualitative, 20–25 μm pore size). The mycelium was rinsed with 0.9% NaCl solution to separate blastospores and remove the residual cultivation medium. The separated mycelium was kept sterile and chilled on ice until further use.

The blastospore concentration of the collected filtrate was determined using a cell counting chamber (Thoma new, Paul Marienfeld GmbH & Co. KG, Lauda-Königshofen, Germany). For PVA film and coating experiments, the required amount of blastospores, and thus filtrate volume was transferred into 50 mL tubes and centrifuged at 4 °C and 5252×*g* for 15 min (Rotixa 50RS, Hettich GmbH & Co. KG, Tuttlingen, Germany). The supernatant was removed and the pellet was washed with sterile 0.9% NaCl once, before being centrifuged again. After the supernatant was removed, the pellet was resuspended in the respective PVA coating solution, depending on the experiment.

Conidia for PVA film experiments were derived from freeze-dried biomass cultivated on PDA at 25 °C for 7 days. Conidia were harvested from PDA plates with a sterile aqueous solution of 0.1% Tween 80®, the concentration determined by cell chamber counting and finally prepared as previously described for blastospores, but without the additional washing step.

### Preparation of polyvinyl alcohol solutions and films

The evaluation of different PVA based on their impact on blastospore release and survival was investigated on thin films.

PVA used in this study are Mowiol® 4-88 (~ 31,000 g/mol), Mowiol® 8-88 (~ 67,000 g/mol) and Mowiol® 10-98 (~ 61,000 g/mol). Here the first number represents the viscosity of a 4% aqueous solution at 20 °C which is derived from its molecular weight. The second number indicates the degree of hydrolysis of the polyvinyl acetate, where 98% is considered as a fully hydrolyzed grade.

Thin PVA films were prepared as follows. The final concentration of PVA in the filmogenic solutions was 4%, regardless whether they were pure or blended. To prepare pure PVA solutions either PVA 4-88, 8-88 or PVA 10-98 was thoroughly mixed with ultra-pure water and finally dissolved by heating at 121 °C for 15 min. The different PVA/PEG/Lec blends were prepared by first mixing all solid ingredients i.e. either PVA 4-88, PVA 8-88 or PVA 10-98 with 2.0% PEG 4000 and 0.8% Soy lecithin followed by adding ultra-pure water to reach the final concentrations. Each mixture was thoroughly blended and finally dissolved and sterilized by heating at 121 °C for 15 min (it should be noted that heating increases crystallinity (Wong and Parasrampuria 1996), thus, decreases solubility, which we however accepted in favor of sterility and process time).

Finally, the cooled solutions were added to the prepared *M. brunneum* blastospore or conidia biomass (see “Microorganisms, culture conditions and preparation of biomass”) and pellets resuspended, resulting in 1 × 10^7^ spores/mL.

Thin films were casted by dispensing 500 µL of the PVA solutions or PVA blends into prefabricated 25 mm wells of silicone mats, and drying to constant weight at 60 °C for 20 min and at 40 °C for 35 min in a ventilated drying cabinet (Memmert GmbH + Co.KG, Schwabach, Germany). After demonstrating that conidia (as benchmark) and blastospores survived equally in water-soluble PVA films (Additional file 1: Fig. S1), making blastospores a promising active ingredient, we subsequently examined the suitability of different PVA types for blastospore survival and release. Constant weight of the different PVA films was previously checked by re-drying the films at 90 °C for 60 min and re-weighing. The thin films were evaluated for the release rate and drying survival of *M. brunneum* blastospores.

### Bead production

Calcium alginate beads were prepared with sterilized materials under aseptic conditions and as previously described (Hermann et al. 2021). For the preparation of the encapsulation suspensions listed below, pre-dissolved sodium alginate was used, which was produced by dissolving sodium alginate (CEROGA Sodium Alginate Type NA5030, C.E. Roeper GmbH, Hamburg, Germany) in ultra-pure water to a final concentration of 4.0% and autoclaving for 6 min at 121 °C. During preparation, the encapsulation suspensions and beads that contained fungal biomass were chilled on ice until further use.

### Calcium alginate/starch suspension

To prepare the encapsulation suspension for calcium alginate/starch beads (hereafter referred to as starch beads), 2.0% sodium alginate and 20.0% native corn starch (CIF GmbH, Siegburg, Germany) were thoroughly mixed at room temperature for 10 min until the starch was completely suspended. Finally, ultra-pure water was added to reach the final concentrations.

### Attract-and-kill suspension

To prepare the encapsulation suspension for AK beads, first, 2.0% sodium alginate and 16.7% *S. cerevisiae* were mixed at room temperature for 5 min. Then, 20.0% native corn starch was slowly added and blended for 10 min. Subsequently, 1.0% *M. brunneum* mycelial biomass was added and the mixture was gently stirred for 2 min. Finally, ultra-pure water was added to reach final concentrations.

### Bead formation and drying

Beads were produced by dripping the respective encapsulation suspension with a sterile syringe with cannula (0.90 × 40 mm Sterican, B. Braun Melsungen AG, Melsungen, Germany) by means of a syringe pump (Cole-Parmer, Vernon Hills, USA) into a cold sterile 180 mM CaCl_2_ solution while stirring. The pumping speed was set to 4 mL/min. Beads cured for 20 min before being separated from the gelation bath and rinsed with ultra-pure water. Drying was performed in two steps. First, moist beads were dried under a laminar flow at room temperature [23 ± 2 °C, approx. 30% relative humidity (RH)] for 24 h and subsequently further dried in a self-constructed fluidized bed dryer at 37 ± 2 °C and approx. 10% RH for 10–15 min until the desired water activity of 0.2–0.3 was reached, unless stated otherwise. The water activity was monitored with a water activity meter (LabMASTER-aw, Novasina AG, Lachen, Switzerland) at 25 °C. Water activity and temperature stable times were set to 2 min and 1 min, respectively.

### Coating

Pre-dried starch beads or AK beads were coated with PVA/PEG/Lec containing 4.16 ± 0.62 10^7^ BS/mL blastospores in a self-constructed lab scale rotating drum dryer. Control starch beads were also coated with PVA/PEG/Lec but without blastospores. The process parameters are listed in Table 1.

**Table 1:** Process parameters during coating with a self-constructed drum dryer

Parameter	Unit	Value
Drum diameter	cm	16.0
Drum speed	rpm	14.0
Drum load	g	> 25.0
Airflow temperature^a^	°C	55.0
Bulk temperature	°C	35.0
Nozzle diameter	mm	0.3
Atomizing air	bar	1.2
Spray rate	mL/min	80.0

^a^Above the bulk during coating

We developed the following formula to approximate the required amount of coating suspension ($V$) based on the drum load and the desired coating thickness as follows

$$V=\frac{{A}_{s}\cdot m\cdot l}{f}\cdot \frac{10}{9},$$

where ${A}_{s}$ represents the specific surface area of one kilogram beads, $m$ is the drum load, $l$ is the desired coating thickness (dry), which was 15 µm, and $f$ is the proportion of dry matter, mainly PVA, of the dried solution, which was previously determined. In addition, the loss of the coating solution during spraying was estimated and included at 10%.

After the coating process, coated beads were further dried in a self-constructed fluidized bed dryer at 37 ± 2 °C and approx. 10% RH for 10 min.

### Determination of blastospore release rate from polyvinyl alcohol films

To assess the solubility of the different PVA, and thus, the release of fungal spores, dry PVA films were dissolved in 1 mL 0.9% NaCl and the blastospore concentration of the same sample was measured after 1, 3, 5, 10, 20, 30, 60, 90 and 180 min using a cell counting chamber. The decreasing sample volume was taken into account when calculating the blastospore concentration. Blastospores from pooled permeate were divided among the treatments. For each treatment, five films were dried sequentially, with treatments dried in parallel. Each dissolving film was measured repeatedly. The experiment was conducted once. Only films from pure PVA were examined for blastospore release, as the residues of the PVA/PEG/Lec blends interfered with accurate counting of blastospores.

### Determination of blastospore survival in polyvinyl alcohol films

The blastospore survival was evaluated by determining the colony forming units (CFU) using a standard plate counting method on 39 g/L semi-selective PDA supplemented with 0.05 g/L cycloheximide, 0.1 g/L streptomycin, 0.05 g/L tetracycline and 100 mg/L dodine, modified according to Strasser et al. (1996). Dry films were dissolved in 1 mL 0.9% NaCl for 90 min. Blastospores or conidia suspended in 0.9% NaCl or 0.1% Polysorbate 80 (Tween®80), respectively, were used as positive controls, whereas PVA and PVA blends without fungal spores were used as negative controls. Plates were cultivated at 23 °C for 14 days in the dark (INCU-Line 150R Premium, VWR International GmbH, Darmstadt, Germany) and checked daily. Colonies were counted when clearly visible. Blastospores from pooled permeate were divided among treatments. For each treatment, five films were dried sequentially, with treatments dried in parallel. Each dissolved film was analyzed three times. The experiment was conducted once.

### Determination of the conidia concentration on beads

The spore forming capacity of encapsulated *M. brunneum* on coated AK beads was determined by counting the conidia formed on the bead surface using a cell counting chamber. Dried AK beads were produced as described previously (see “Bead production”), and beads from the same batch were subsequently coated either with PVA 4-88/PEG/Lec without blastospores, or with sterile demineralized water, as control, in order to evaluate the impact of the coating layer but not the coating process. Beads were rehydrated and incubated on 1.5% water agar in sealed Petri dishes at 23 °C in the dark for 3 weeks. The conidia concentration was determined weekly. At each sampling time point, 15 coated or control beads were analyzed. Beads were transferred into 1 mL 0.1% Tween®80 and thoroughly mixed (Vortex Genie, neoLab Migge GmbH, Heidelberg, Germany) for at least 1 min to detach the conidia. The experiment was conducted once.

### Virulence against *T. molitor* larvae

To investigate whether blastospores coated in PVA were still virulent and to evaluate the overall effect of the water-soluble blastospore coating, a bioassay against *T. molitor* larvae (obtained from a pet shop, Bielefeld, Germany) was conducted (adapted from Przyklenk et al. 2017). The PVA suspensions used in the coating process and untreated blastospores were used as controls. Following treatments were investigated: blastospores in 0.9% NaCl as positive control (BS NaCl), the PVA coating suspension with and without blastospores (BS PVA, Control PVA) to investigate the impact of PVA on blastospores and larvae, blastospores detached from coated starch beads (BS detached) to investigate whether detached spores are still virulent, plain starch beads (S) as negative control and sterility testing, starch beads coated with PVA only (S Coat) to check sterility and investigate the impact of the coating material on larvae, starch beads coated with blastospores (S BS) as main treatment to investigate the impact of the blastospore coating, uncoated AK beads (AK) as standard formulation thus benchmark, AK beads coated with blastospores (AK BS) to investigate the impact on the depot (application as intended in the field), and lastly no treatment at all as negative control (Non).

Beads and coating solutions were prepared as previously described (see “Bead production” and “Preparation of polyvinyl alcohol solutions and films”). Based on the research preceded in this study, beads were coated with the PVA 4-88/PEG/Lec mixture only. For treatments S BS and AK BS, 45 g starch beads and 62 g AK beads (correspond to the same calculated total surface area) were both coated with 60 mL of the same coating solution containing 4.16 × 10^7^ ± 0.62 BS/mL.

The bioassay was performed by incubating individual larvae, similar in size [25.06 ± 2.14 mm, ~ 13–15th instar (Park et al. 2014)], in culture dishes (60 × 15 mm, non-treated, STARLAB International GmbH, Hamburg, Germany) on cellulose filters (No. 401, 55 mm, qualitative, 12–15 µm pore size, VWR International GmbH, Darmstadt, Germany). First, cellulose filters were moistened with 200 and 500 µL sterile tap water before liquid or solid treatments (including negative control ‘non’) were applied, respectively. Subsequently, either 500 µL of each suspension (Control PVA, BS NaCl, BS PVA, BS detached) or 5 beads of each bead formulation (S, S Coat, S BS, AK, AK BS) were inoculated. Then, larvae were washed with sterile tap water, placed in the culture dishes and fed with three sterile oat flakes. The treatments Control PVA, BS PVA and BS detached were performed with 40 larvae each (two per culture dish). The remaining treatments were each performed with 20 larvae (one per culture dish). All 200 samples were incubated at 25.1 ± 0.1 °C and 83 ± 2% RH in the dark for 12 days. Temperature and humidity were monitored with a humidity/temperature logger (ebro EBI 20, Xylem Analytics Germany Sales GmbH & Co. KG, Weilheim, Germany). Every second day, filter papers were moistened with 100 µL sterile tap water and oat flakes were replaced with fresh ones to prevent mold. Samples were checked daily for dead individuals. Dead individuals were first immersed in 70% (v/v) ethanol for five seconds, then washed in sterile tap water and finally placed on semi-selective PDA for analysis of *M. brunneum* caused mycosis. Petri dishes were sealed with Parafilm® M (Pechiney Plastic Packaging Inc., IL, USA) to prevent evaporation and then incubated at 25 °C in the dark. After 14 days, cadavers were examined for infection by *M. brunneum* based on morphological characteristics, such as white mycelium with olive-green conidia packages (see Fig. 5g, h). When individuals showed unusual fungal morphology (fluffy, grayish mycelium and/or black spores), indicating mold infestation, or were covered presumably with different fungi, the respective mycelia/conidia were scraped off and frozen for further examination, i.e. DNA extraction (see Additional file 1: Fig. S5).

Starch and AK beads with and without BS coating, which were used for the bioassay, were incubated on 1.5% water agar at 25 °C for 14 days to check for contaminations and conidiation. The conidia concentration on 10 beads per treatment was determined using a cell counting chamber. Beads were transferred into 1 mL 0.1% Tween®80 and thoroughly mixed for at least 1 min to detach the conidia. Additionally, the total blastospore concentrations (cell chamber counting) and CFU of the following treatments were determined as controls: the detached coating from starch (BS detached) and AK beads, the BS NaCl suspension, the BS PVA suspension as well as the NaCl solution and the PVA solution as negative controls. The entire bioassay was conducted once.

### Scanning electron microscopy

For analyzing the outer bead and coating morphologies, scanning electron micrographs (SEM) were captured from plain starch beads as well as starch beads coated with and without blastospores which were previously used for the virulence bioassay (see “Virulence against T. molitor larvae”). Beads were sputtered with gold (EM SCD005, Leica Microsystems, Wetzlar, Germany) prior to SEM (S-450, Hitachi, Tokyo, Japan).

### Statistical analysis

Statistical analyses were conducted with SPSS Statistics v. 27 (SPSS, Chicago, USA) with a significance level of *P* < 0.05. All data are presented as mean values ± standard deviations (SD). Normality and homogeneity of variance were analyzed with Shapiro–Wilk’s test/Q–Q-Plots and Levene’s test, respectively. To analyze the influence of treatments or the influence of treatment and time, one-way analysis of variance (ANOVA) or two-way repeated measures (RM) ANOVA with Bonferroni post hoc tests (Bon) were conducted for normally distributed and homogeneous residuals. Welch’s correction was applied for nonhomogeneous variances and Games-Howell post hoc conducted. The latter was also conducted when samples were unequal in size. The sphericity of the matrix assumption was assessed with the Mauchly sphericity test and F values were corrected accordingly using the Greenhouse–Geisser approach. For non-normally distributed sets, Kruskal–Wallis tests with multiple comparisons between groups were calculated. Means of two groups were compared by two-tailed t-test. When testing for constant weight of dried PVA films, paired-samples Equivalence test was conducted with a lower limit of 3% (Minitab 19, State College, USA). The survival probability of *T. molitor* larvae were analyzed by Kaplan–Meier and compared with log-rank test (Mantel-Cox) for both global and pairwise differences.

## Results

### Blastospore release depended on  polyvinyl alcohol type

To analyze the suitability of different PVA, resulting films were examined regarding their blastospore release, and thus, water solubility, as well as blastospore survival. It was also verified that the PVA films were dried to a constant weight to better evaluate the results of the survival experiment. Furthermore, we examined whether PEG and Lec as plasticizer and emulsifier, respectively, enhance blastospore survival in thin PVA films.

All PVA films were dried to a constant weight except for PVA 10-98 (Additional file 1: Fig. S2). The remaining water content after standardized drying tended to increase with increasing molecular weight but mainly with increasing degree of hydrolysis, while it seemed not to be affected by PEG and Lec (Additional file 1: Fig. S3).

**Fig. 1:**
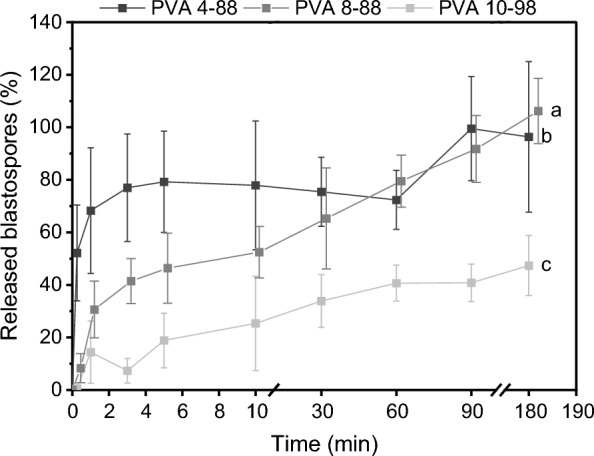
Blastospores detached significantly faster from polyvinyl alcohol (PVA) films 4-88 and 8-88 compared to PVA 10-98. Dried PVA films were immersed in 0.9% NaCl and the concentrations of detached blastospores were determined by cell chamber counting. Different letters indicate differences according to two-way RM ANOVA with Bonferroni Post-hoc (n = 5)

The blastospore release rate was determined via cell chamber counting only for pure PVA types, due to impurities in the PVA blends caused by Lec. It was found that time (F_2.2,26.4_ = 45.95, *P* < 0.001) and the interaction of time and treatment (F_4.4,26.4_ = 4.4, *P* = 0.006) had a significant impact on the blastopore release (Fig. 1).

Within the first 5 min, the blastospore release rate quadrupled as the molecular weight and degree of hydrolysis decreased (PVA 4–88: 15.9 ± 3.9%/min, PVA 8–88: 9.3 ± 2.7%/min, PVA 10–98: 3.8 ± 2.1%/min), consequently, most blastospores detached from PVA 4–88 at 79 ± 19% (F_2,14_ = 13.7, *P* = 0.001, Bon, *P* < 0.039). After 5 min, the release rate decreased sharply for all PVA types (PVA 4–88: 0.1 ± 0.2%/min, PVA 8–88: 0.3 ± 0.0%/min, PVA 10–98: 0.2 ± 0.1%/min). Only 48 ± 1% of the immobilized blastospores detached from PVA 10–98 after 180 min. Moreover, PVA 10–98 films did not dissolve completely; residues were still visible after 180 min, in contrast to the lower degree of hydrolysis films. For comparison, approximately 50% of blastospores detached from PVA 4-88 and PVA 8-88 already within 15 s and 5 min, respectively.

### Adjuvants improved blastospore survival in polyvinyl alcohol films

Next, we investigated how the PVA type (molecular weight/viscosity and degree of hydrolysis/crystallinity) influences the blastospore survival in thin films.

**Fig. 2:**
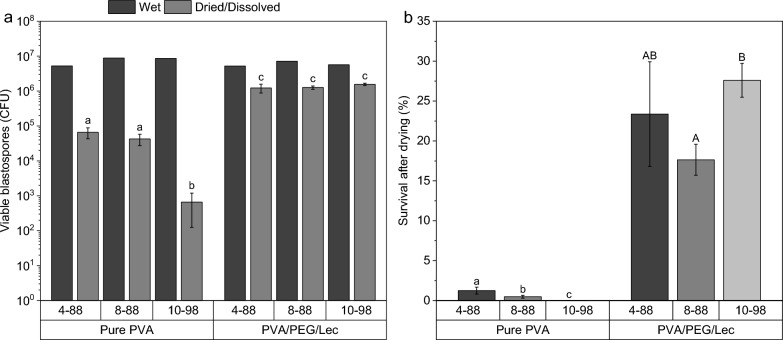
Blastospore (BS) viability after drying/dissolving was significantly reduced in pure polyvinyl alcohol (PVA) 10-98, but adding polyethylene glycol 4000 (PEG) and soy lecithin (Lec) significantly enhanced blastospore viability in all PVA types (**a**). The blastospore survival significantly decreased depending on the molecular weight and degree of hydrolysis of pure PVA, but PEG and Lec counterbalanced these effects (**b**). Dried PVA films were immersed and dissolved in 0.9% NaCl and CFU determined via standard plate counting on semi-selective PDA. Sealed Petri dishes were cultivated at 23 °C for 14 days and checked daily for CFU. Different letters above bars indicate significant differences according to one-way ANOVA with Games Howell post-hoc n = 5 for dried/dissolved samples, and with Bonferroni post-hoc n = 5 for survival. Upper and lower case letters refer to each other only

As expected, the CFU concentrations decreased due drying/dissolving in all PVA types, but strongest in pure PVA 10-98 (Fig. 2a). In pure PVA 10-98 films, significantly fewer blastospores were viable after drying/dissolving, even considering its lower solubility and blastospore release. However, PEG and Lec mitigated this negative effect and significantly improved blastospore viability for all PVA types (Welch’s F_5,9.3_ = 176.65, *P* < 0.001).

According to two-factorial ANOVA, the adjuvants PEG and Lec, but not the PVA type or their interaction, significantly influenced the blastospore viability (F_1,24_ = 391.1, *P* < 0.001 and F_2,24_ = 1.7, P = 0.205 and F_2,24_ = 3.4, *P* = 0.050, respectively). In pure PVA films, blastospore survival was generally very poor and, moreover, decreased significantly close to zero with increasing molecular weight and degree of hydrolysis (F_2,14_ = 50.96, *P* < 0.001, Fig. 2b). By adding PEG and Lec, surprisingly, most spores survived in PVA 10-98 (27.6 ± 2.12%, F_2,14_ = 6.38, *P* < 0.013). The water activities of dried films, with or without PEG/Lec ranged between 0.189 and 0.202.

### Polyvinyl alcohol formed a uniform layer on beads

**Fig. 3:**
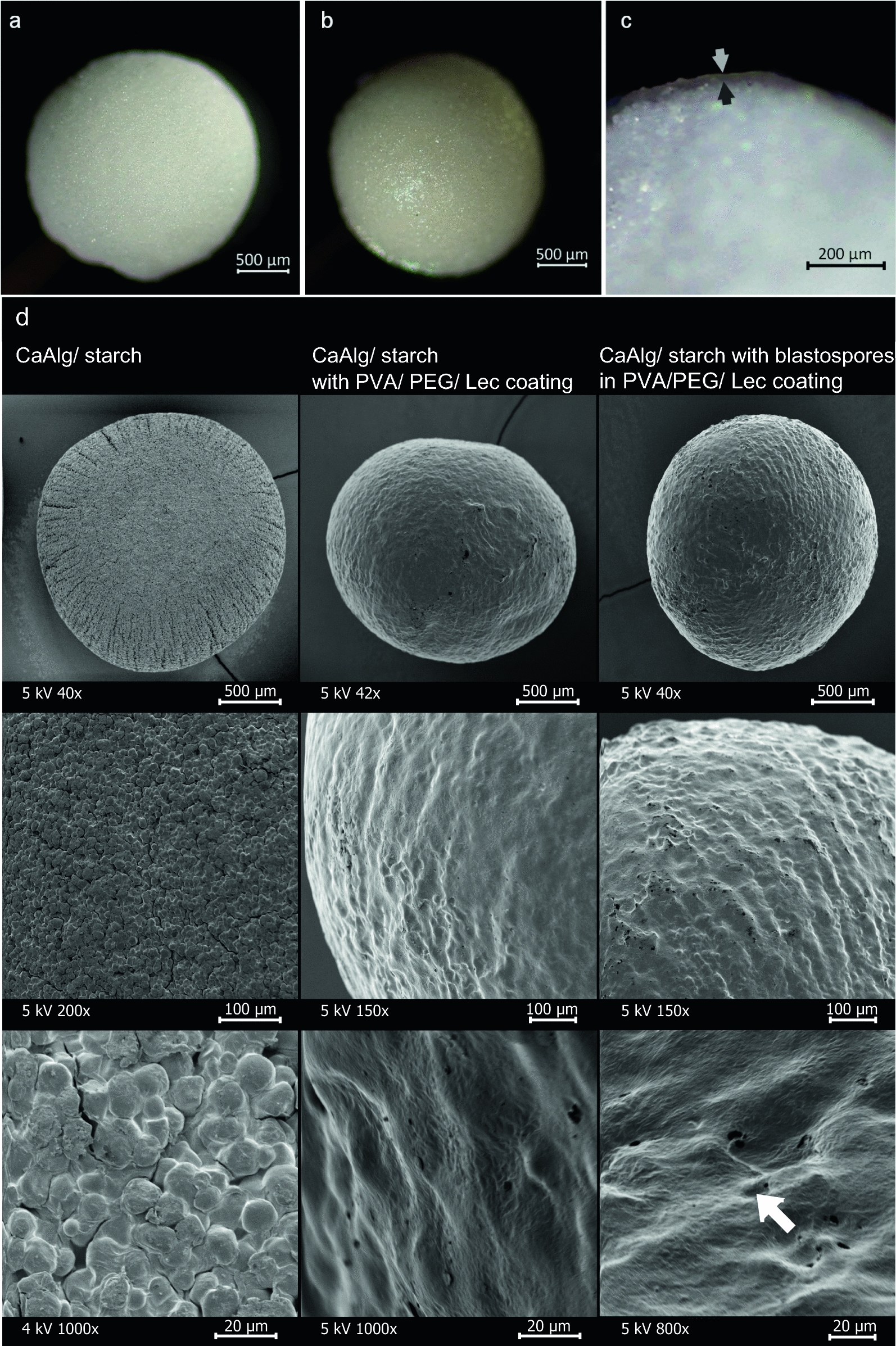
Polyvinyl alcohol (PVA) formed a thin film on the calcium alginate beads with embedded blastopores. Light microscopic images of an uncoated (**a**) and with PVA 4-88/PEG/Lec coated starch bead (**b**, **c**). Grey arrows indicate the film layer on the bead with a thickness of 22.4 ± 7.3 µm (n = 20). SEM images (**d**) of a calcium alginate/starch bead (CaAlg/starch, left column), coated with PVA 4-88/polyethylene glycol/lecithin (PVA/PEG/Lec, middle column) or coated with blastospores in PVA/PEG/Lec (right column) in three different magnifications (rows). The white arrow presumably indicates a single, germinated blastospore adhering to the coating film. Most of the blastospores must be embedded in or under the coating layer, as 110 blastospores would be expected in the image section shown (bottom rightmost). Beads were sputtered with gold prior to SEM

The homogeneity and integrity of the coating material PVA 4-88/PEG/Lec was investigated by light microscopy and SEM. The coating resulted in a thin PVA layer with a coating thickness of 22.4 ± 7.3 µm. Beads appeared fully and evenly coated without visible cracks or brittle spots (Fig. 3). Moreover, the coating completely dissolved in water within a few seconds, visualized by previously staining the coating with ink (Additional file 1: Fig. S4). The SEM images confirmed that the PVA formed a thin, uniform film on the beads, covering the rough surface caused by the starch particles. As a result, the coated beads presented a smoother surface (Fig. 3). SEM of beads used in the bioassay also revealed that blastospores appear to be embedded within or under the coating layer, as approximately 110 blastospores would be expected in the image section shown (Fig. 3d, bottom rightmost).

### Polyvinyl alcohol coating maintained conidiation on attract-and-kill beads

To assess whether the coating—but not the coating process—affects growth and sporulation of the encapsulated *M. brunneum*, dried AK beads were coated with PVA 4-88/PEG/Lec or with water as control.

**Fig. 4:**
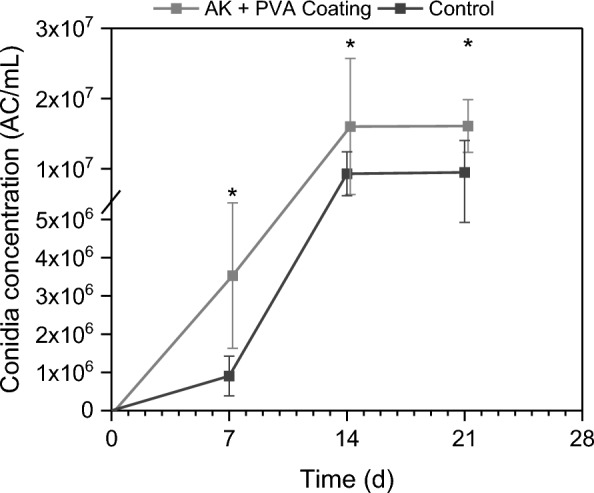
The polyvinyl alcohol (PVA) coating increased the conidia concentration on attract-and-kill (AK) beads compared to control AK beads. AK beads were coated either with PVA 4-88/PEG/Lec or water as control and subsequently dried. Beads were cultivated on 1.5% water agar in sealed Petri dishes at 23 °C in the dark for 3 weeks. Formed conidia were washed from the beads and the concentration determined by cell counting. Asterisks indicate significant differences between coated AK beads and control beads at the given time point according to t-test (n = 15)

Surprisingly, the PVA 4-88/PEG/Lec coating significantly increased the conidia concentration on AK beads at each of the measured time points (T(16.1) = 4.986, *P* < 0.001; T(16.9) = 2.468, *P* = 0.025; T(28) = 4.170, *P* < 0.001), Fig. 4) compared to the processed control. The coating accelerated the conidia formation, and thus, the conidia concentration tripled after 7 days and finally increased by 70% after 21 d. Although treated exactly the same, water activities after drying differed slightly, but in favor of control beads as more cells survive when dried less; a_w_ (control) = 0.42 and a_w_ (coated) = 0.31.

### Blastospore coating accelerated the mortality of *T. molitor* larvae

To evaluate the effectiveness of the developed blastospore coating, a bioassay was performed with *T. molitor* larvae. For this purpose, the PVA suspensions and coated bead formulations were investigated.

After coating, approximately 47% and 39% of the mathematically possible blastospores from the coating solution adhered to starch and AK beads, respectively. Accordingly, the total blastospore concentrations were slightly elevated on starch beads compared to AK beads, but the exact opposite was true for CFU (Table 2). Consequently, survival of blastospores adhering to starch and AK beads differed significantly (21% and 65%, respectively, T_10.87_ = − 8.396, *P* < 0.001). Approximately 87% of the blastospores in the coating stock solution were vital. By normalizing the blastospore survival on beads to this very viability in the stock solution, a final survival of 24% on starch beads and 75% on AK beads was calculated. It should be noted that CFU were determined on semi-selective PDA supplemented with antibiotics and fungicides which decelerate growth and eliminate weakened blastospores, consequently, lowering the survival, but also reflecting the ratio of vigorous, presumably virulent blastospores. In all treatments, the blastospores and also the encapsulated mycelium were viable, as shown by subsequent conidia formation after 14 d (Table 2). However, the conidia concentration on coated AK beads was 18% lower compared to uncoated AK beads. Considering the previous results (see “Polyvinyl alcohol coating maintained conidiation on attract-and-kill beads” ), which demonstrated that the coating layer did not negatively impact sporulation, this reduced sporulation can be attributed to the coating process induced stress.

**Table 2:** Survival obtained from total blastospore concentrations and viable blastospore concentrations of the blastospore suspension and on the coated bead formulations determined by cell chamber counting and standard plate counting, respectively

Treatment	Blastospore survival normalized^a^	Total blastospore concentration n = 5–10	Viable blastospore concentration n = 4–10	Conidia concentration after cultivation n = 10
Blastospore suspensions	(%)	(10^7^ BS/mL)	(10^7^ CFU/mL)	
BS NaCl	n/a	1.18 ± 0.11	1.93 ± 0.17	–
BS PVA	n/a	0.76 ± 0.08	0.90 ± 0.20	–
BS detached	31 ± 23	0.80 ± 0.14	0.23 ± 0.19	–
Coating stock solution	100 ± 23	4.16 ± 0.62	3.64 ± 1.01	–

Bead formulations	(%)	(10^5^ BS/bead)	(10^5^ CFU/bead)	(10^7^ conidia/bead)
Starch BS	24 ± 5	1.20 ± 0.22	0.34 ± 0.28	0.05 ± 0.03
AK BS	75 ± 16	1.00 ± 0.19	0.64 ± 0.20	2.09 ± 0.48
Starch	–	–	–	0.00 ± 0.00^b^
AK	–	–	–	2.56 ± 0.69

^a^Data were normalized to the blastospore viability in the coating stock solution, n/a: not applicable (no relation to the stock solution)

^b^Conforming sterility

**Fig. 5:**
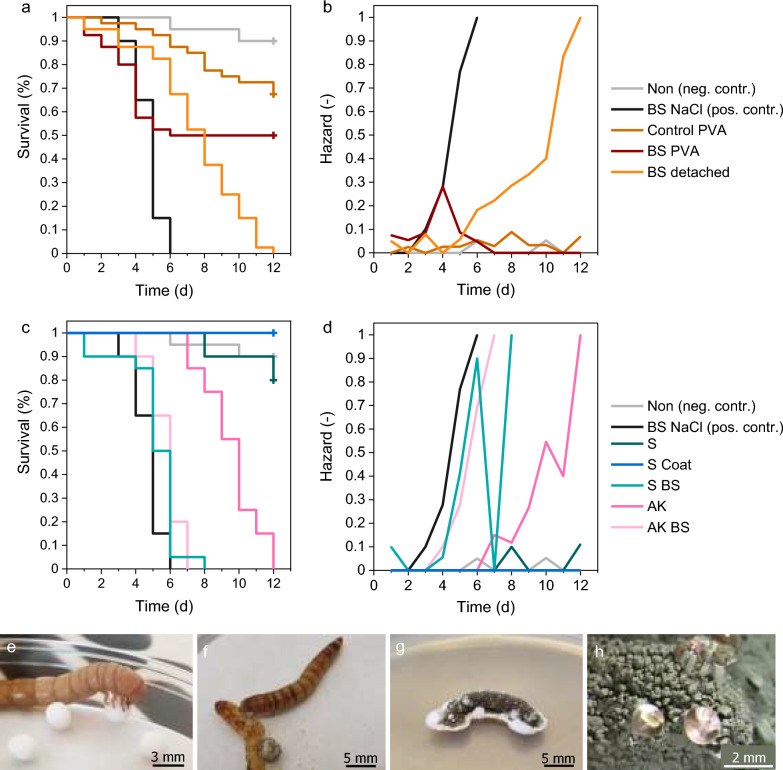
Cumulative survival of *Tenebrio molitor* (**a**, **c**) and corresponding hazard (**b**, **d**) 12 days after exposure to control suspensions (**a**, **b**) and bead formulations (**c**, **d**). Polyvinyl alcohol 4-88/polyethylene glycol/lecithin coating suspension with and without blastospores (BS PVA, Control PVA), blastospores detached from coated starch beads (BS detached), plain starch beads as negative control (S), starch beads coated with PVA only (S Coat) and starch beads coated with blastospores (S BS), uncoated AK beads (AK), AK beads coated with blastospores (AK BS), blastospores in 0.9% NaCl as positive control (BS NaCl) and no treatment at all as negative control (Non). Individuals were right-censored if they were still alive at the end of the experiment. Censoring is indicated by tick marks on the Kaplan–Meyer curve. *T. molitor* larva immediately fed on starch beads (**e**), and presumably molted after sporulated attract-and-kill bead attached to its skin. The bead was still attached to the molt (**f**). The sporulation of *Metarhizium brunneum* CB15-III on infected *T. molitor* larva cultivated on semi-selective PDA after 14 days (**g**) was microscopically analyzed and showed morphological characteristics of *M. brunneum* (**h**)

According to the bioassay with *T. molitor*, as expected, larval survival was 90% in the reference control group and no mycosis visible, whereas mortality with *M. brunneum* infection was 100% when treated with 9.7 × 10^6^ viable blastospores as positive control (Additional file 1: Table S1). When treated with the control PVA coating solution without blastospores, 68% of larvae survived, suggesting that 500 µL 4% coating solution was debilitating and lethal to *T. molitor* larvae (e.g. by immobilization and asphyxiation). Here, one contamination with *M. brunneum* and two with unknown fungus were confirmed by PCR (Additional file 1: Fig. S5). The PVA solution containing blastospores was more lethal, yet 50% of larvae still survived, indicating that the virulence of blastospores was attenuated. In contrast, blastospores which were detached from coated starch beads were as lethal as the positive control, and 95% of larvae were dead and infected. The blank starch-formulation had no effect and was non-toxic, but one single contamination with *M. brunneum* was confirmed by PCR (Additional file 1: Fig. S5). Remarkably, 100% of larvae survived when treated with coated starch beads (without blastospores), confirming that the PVA coating is non-toxic to larvae. Starch beads and AK beads both coated with blastospores, as well as regular AK beads, were highly lethal and 95–100% of larvae were infected with *M. brunneum.* However, the key difference between these formulations was the speed of action, as there were significant differences in the *T. molitor* mortality rate between treatments (χ^2^(9) = 208, *P* < 0.001, Fig. 5a, c). The blastospore coating accelerated the kill effect of AK beads. The time needed to induce mortality of 50 or 75% (LT_50,_ LT_75_) was reduced by 40% (Table 3), and consequently, the blastospore coating was almost as effective as the positive control. Accordingly, the survival of larvae treated with coated and uncoated AK beads differed significantly (χ^2^(1) = 36.2, P < 0.001). Referring to LT_50_, interestingly, blastospore-coated starch beads performed slightly faster than blastospore-coated AK beads. However, the survival of larvae did not differ significantly (χ^2^(1) = 1.03, *P* = 0.31), which furthermore indicates that the larval mortality was induced by the immobilized blastospores in the coating. The encapsulated fungus could not exert its effect in this scenario with a limited number of larvae because the blastospore coating killed all larvae beforehand. Moreover, although fewer blastospores survived on coated starch beads than on coated AK beads (− 47%), both were equally effective. Accordingly, a dose of 1.7 × 10^5^ viable blastopores (3.4 × 10^4^ blastospores/bead) was sufficient to rapidly kill the larvae. In contrast, uncoated AK beads had the latest onset of effect and acted significantly slower compared to all blastospore treatments, excluding treatment BS PVA (*P* < 0.012).

**Table 3:** Different lethal times for the different control suspensions and bead formulations according to log-rank test (MV ± SE)

Treatment	LT_25_ (d)	LT_50_ (d)	LT_75_ (d)
BS NaCl (pos. ctrl.)	4.0 ± 0.4	5.0 ± 0.2	5.0 ± 0.2
BS PVA	4.0 ± 0.4	6.0 ± 0.0	–
Control PVA	9.0 ± 2.0	–	–
BS detached	6.0 ± 0.5	8.0 ± 0.5	9.0 ± 0.6
AK	8.0 ± 0.6	10.0 ± 0.3	10.0 ± 0.5
AK BS	5.0 ± 0.4	6.0 ± 0.2	6.0 ± 0.2
S BS	5.0 ± 0.3	5.0 ± 0.3	6.0 ± 0.1
S Coat	–	–	–
S	–	–	–
Non	–	–	–

Blastospores in 0.9% NaCl as positive control (BS NaCl), polyvinyl alcohol 4-88/polyethylene glycol/lecithin coating suspension with and without blastospores (BS PVA, Control PVA), blastospores detached from coated starch beads (BS detached), uncoated AK beads (AK), AK beads coated with blastospores (AK BS), starch beads coated with blastospores (S BS), starch beads coated with PVA only (S Coat), plain starch beads as negative control (S), and no treatment at all as overall negative control (Non)

For the first 4 days, blastospores in the PVA/PEG/Lec suspension (BS PVA) acted even faster than the blastospores in NaCl (positive control), although the viable blastospore concentration was slightly lower (see Table 3). This further indicates that the PVA blend weakened the larvae. However, after 4 days, hazard values declined to zero, thus larval survival remained constant at 50 % until the end of the experiment, indicating that the blastospores had lost their virulence. Inconsistencies within this treatment became apparent as either both larvae in the respective culture vessel died or none did.

The blastospores which were detached from starch beads prior to application (‘BS detached’) performed significantly slower than those in every other blastospore treatment (*P* < 0.004), possibly due to the lower viable blastospore concentration. However, in contrast to the blastospores in the PVA/PEG/Lec coating solution, detached blastospores remained virulent over the entire test period and killed all larvae. Nevertheless, it should also be noted that within this period virulent conidia can naturally be formed originating from blastospores.

However, there was a significant difference in effect compared to the AK treatment, in which the fungus first needs to grow out and form conidia to take effect (χ^2^(1) = 6.48, *P* = 0.011).

There were no significant differences between the untreated reference group and all blastospore-free negative controls (S, S Coat and Control PVA, *P* = 0.06–0.42).

All fungal treatments caused a significantly increased risk of larval mortality in respect to the reference control group (*P* ≤ 0.002), also portrayed by increased hazard values (Fig. 5b, c).

It is worth mentioning that *T. molitor* larvae fed on beads, and molted quite frequently, for instance after sticking to beads which were covered in conidia (Fig. 5e–h). Gel electrophoresis of extracted DNA confirmed *M. brunneum* CB15-III infection apart from the above mentioned contaminations (Additional file 1: Fig. S5).

## Discussion

Our results indicate that the immediate release of virulent blastospores from an appropriate, water-soluble coating improves the effectiveness of AK beads.

The release rate of blastospores from the PVA coating depended on the PVA’s degree of hydrolysis and molecular weight. For low molecular weight and degree of hydrolysis, most blastospores immediately detached from the coating. Although the survival of blastospores after drying also depended on the PVA’s molecular weight and degree of hydrolysis, the addition of PEG and Lec had a much greater positive impact on survival. Furthermore, when applied as coating on alginate beads, PVA/PEG/Lec maintained sporulation of the encapsulated fungus and possibly protected it during the process-related rehydration-drying-cycle compared to the processed control. Eventually, the blastospore coating of AK beads accelerated the mortality of *T. molitor* larvae.

### Active ingredients in base bead and coating

The fermentation of mycelium and blastospores is preferred because it is cheaper and more efficient than that of conidia (Jaronski and Mascarin 2017). Moreover, selecting the appropriate form of inoculum is important for efficacious pest control. Blastospores have a high infectivity in insects and are therefore considered more virulent (Hall 1979; Miranpuri and Khachatourians 1990; Wang et al. 2013; Alkhaibari et al. 2016, 2017; Yun et al. 2017). Although conidia are naturally more resistant to heat stress and lack of moisture (Wyatt et al. 2013), in the present study, conidia and blastospores did not significantly differ in survival after drying in PVA. Therefore, the biotechnological strategy was to take advantage of the entire biomass of submerged cultivation, i.e. mycelia and blastospores, for the production of coated AK beads, thereby optimizing the overall manufacturing process.

### Blastospore release rate

The solubility of PVA depends primarily on its degree of hydrolysis and its viscosity which derived from its molecular weight. With decreasing molecular weight or degree of hydrolysis the water solubility of PVA increases (Wong and Parasrampuria 1996). Accordingly, in the present study, the blastospore release (rate) also increased significantly with decreasing molecular weight and degree of hydrolysis. These results are in accordance with the good water solubility and water vapor absorption from other studies, for instance reported by Vercelheze et al. (2019) and Hwang et al. (2017), respectively. In the study of Hwang et al. (2017), PVA with lowest molecular weight at the same degree of hydrolysis and vice versa, with lowest degree of hydrolysis at the same molecular weight, exhibited the highest water vapor absorption. The fully-hydrolyzed, high-molecular-weight PVA 10-98 which we used in the present study, did not dissolve completely, which is reflected in its low blastospore release. However, fast and complete dissolution of the coating layer is required, mainly to ensure aerobic growth and subsequent sporulation of the encapsulated fungus, as PVA possesses one of the lowest oxygen permeability among polymers (Mokwena and Tang 2012). The oxygen permeability is highly dependent on the overall composition, i.e. degree of hydrolysis, molecular weight, and thus crystallinity (Mokwena and Tang 2012; Hdidar et al. 2017; Idris et al. 2021; Schiessl et al. 2022). Eventually, both events (i) releasing all blastospores for high speed to kill and (ii) ensuring both growth and sporulation of the encapsulated fungus for a long-lasting effect in soil, may ultimately maximize the effect of the formulation.

Since the coating is intended for application in biological crop protection, its biodegradability must be briefly addressed. In general, polymers with simple, saturated hydrocarbon backbones are considered to be poorly or even non-biodegradable, PVA, however, is an exception (Ben Halima 2016; Koltzenburg et al. 2017; Byrne et al. 2021). It is regarded as completely biodegradable, as it can be utilized by some bacteria and fungi as carbon and energy source. Nevertheless, environmental PVA biodegradation is complex, and may occur in some soils at insufficiently low rates (Vaclavkova et al. 2007; Kawai and Hu 2009). Factors that affect its biodegradation are temperature, humidity, oxygen content of the medium and the PVA’s chemical structure and composition (Ben Halima 2016; Koltzenburg et al. 2017). In aqueous aerobic environments, the biodegradation of PVA occurs in line with its water solubility (Chiellini et al. 2003). With regard to the used adjuvants, the degradation of PEG by certain microorganisms has been extensively studied and reviewed. It has been repeatedly found that the molecular weight of PEG strongly influences its biodegradability. The higher its molecular weight, the less degradable it is. However, significant biodegradation of PEG with molecular weights up to 20 kD has also been reported (Cox 1978; Kawai 2002; Chiellini et al. 2003).

### Blastospore survival in polyvinyl alcohol films

In pure PVA films, the blastospore survival after drying and subsequent rehydration decreased significantly with both increasing molecular weight and degree of hydrolysis, but survival was generally very low. Two partially hydrolyzed PVA (88%) and one fully hydrolyzed PVA (98%) were used. Deaker et al. (2007) found that PVA with a degree of hydrolysis of 86.5–89% protected freeze-dried rhizobia better than PVA with higher (98.5%) or lower (78.5–82%) degrees at constant molecular weight. However, material properties responsible for improved survival were not clearly identified due to the complex and disparate nature of the polymers. By adding PEG and Lec, blastospore survival generally improved, and the negative effects related to the molecular weight or degree of hydrolysis were leveled. According to Lim and Wan (1994), adding plasticizers increases the dissolution of PVA films. In the present study, the blastospore release from PVA/PEG/Lec films could not be determined by cell chamber counting due to impurities in the lecithin. However, the adjuvants counteracted the poor release, as supported by CFU values. Moreover, structural changes in the PVA blends may contribute to the increase in survival. For instance, in a study of Lim and Wan (1994), PEG promoted the formation of crevices and pores in the PVA films, which we believe may favor cell survival due to reduced mechanical pressure generally exerted on cells during drying (Chan et al. 2011).

The overall survival of blastospores is in accordance with studies by Lorenz et al. (2020). Here, 14.7% of encapsulated *Metarhizium pemphigi* blastospores survived the drying process in alginate/chitin beads, but at ambient temperature (Lorenz et al. 2020). Considering the relatively harsh but rapid desiccation process in the present study, our results are highly promising, yet providing opportunity for further improvement. A milder drying temperature likely increases blastospore survival, but also prolongs drying and thus overall process time. Considering the envisaged spray coating process, the process temperature affects the PVA’s viscosity and thus possibly the coating result (Zhang et al. 2020). Therefore, investigating desiccation tolerant and robust blastospores (Dietsch et al. 2021) seems highly promising to further enhance the effectiveness of the formulation.

### Technical coating of beads and the impact of adjuvants

Beside its good water solubility and blastospore survival, PVA 4-88 was selected for further investigations, i.e. coating of alginate/starch beads, because it has a lower viscosity than PVA 8-88 at the same concentration. Its lower viscosity makes it more suitable for spray coating, as it is easier to atomize with smaller droplet sizes (Rizkalla and Lefebvre 1975; Andrade et al. 2012), and provides better particle surface coverage (Zhang et al. 2020).

In the present study, the resulting coating layer was relatively thin, even and non-brittle, which is required for controlled release (Azeem et al. 2016). The challenge in developing a technical coating are the high and sometimes contradictory requirements. In the present case, the coating must adhere to the alginate/starch bead and maintain its integrity after drying. Its tensile strength must withstand the normal stresses during packaging, transportation and handling. Only in the soil, after application, should the coating lose its integrity to release the immobilized blastospores. The release in soil is determined by several factors, including temperature, moisture, pH, the presence of salt in the soil as well as the mechanical strength of the coating (Wesołowska et al. 2021). Mechanical strength, in turn, is determined by the coatings composition. In the present study, PEG and Lec were added as adjuvants. PEG is a plasticizer that reduces the brittleness of the film, presumably by increasing the glass transition temperature of the PVA film (Nies and Messing 2006; Falqi et al. 2018). Moreover, the addition of PEG reduces the coatings tensile strength and vapor permeability but increases its elongation at break, flexibility and hydrophilicity (Lim and Wan 1994; Abdel Tawab et al. 2011; Falqi et al. 2018; Ucpinar Durmaz and Aytac 2021). PVA molecules crosslink with the plasticizer via hydrogen bonding, thereby reducing the PVA’s degree of crystallinity, and thus increasing its solubility. Here, the molecular weight and concentration of the plasticizer affect its effectiveness (Sakellariou et al. 1993; Lim and Wan 1994). Jordan and Taylor (2002) found PEG with a higher molecular weight greater than 1000 g/mol to be useful for PVA-based film coatings, but 3000 g/mol was preferred. However, according to Sakellariou et al. (1993), the plasticization of PVA by PEG is inversely proportional to its molecular weight, probably due to phase separation at higher molecular weights. In the present study, PVA was additionally blended with Lec, which is an emulsifier and surfactant with homogenizing, stabilizing and hydrating properties. Interestingly, Jordan and Taylor (2002) noted that Lec also acts as a detackifier after spray coating.

### The coating’s impact on conidia formation on beads

During the coating process, dried beads undergo a cycle of rehydration and drying, which affects the viability of the encapsulated fungus—as found later in the virulence experiment when comparing the conidia concentration of coated and uncoated AK beads. Therefore, of greater interest was, whether the PVA coating layer as such affected the fungus viability and thus conidia formation. Contrary to our assumptions, the conidia concentration not only remained but even increased significantly when beads were coated with PVA 4-88/PEG/Lec compared to ‘water-coated’ beads. There are mainly two explanations: (i) The coating material protected the encapsulated fungus from the process induced stress, or (ii) PVA, PEG and/or Lec supported sporulation itself.

Several microorganism have been reported to be capable of degrading PVA (Ben Halima 2016), however, to the best of our knowledge, there is no study suggesting that PVA supports growth. In contrast, PEG was reported to promote mycelial growth and conidiation of alginate-encapsulated *Trichoderma harzianum* and *B. bassiana*, respectively. The authors even claimed that it may improve the efficacy of biocontrol (Knudsen et al. 1991). The exact mode of action is unexplained, and was also not in the scope of the present study. However, osmotic regulants such as PEG are generally used to control water availably, and thus cell membrane disruption (Potts 1994).

Regarding the influence of Lec, Hofer et al. (2018) identified Lec as the key material attribute of crude soybean oil, which positively affects growth and productivity of some filamentous fungi when low dosed (Hohl 1975; Jones and Porter 1998; Hofer et al. 2018). Therefore, it seems likely that Lec also promotes growth and sporulation of *M. brunneum*.

### Virulence against *T. molitor* larvae

The PVA-based blastospore coating significantly accelerated the mortality rate of *T. molitor* larvae by releasing virulent blastospores, and was consequently able to bridge the time until the uncoated AK beads began to take effect. By the time the AK beads began to take effect, the blastospore-coated bead treatments had already killed all larvae. Moreover, the blank PVA/PEG/Lec coating on beads was proven to be non-toxic to *T. molitor*. However, when directly applying the PVA coating suspension, our findings are not entirely consistent. The PVA suspension affected both larval viability and fungal virulence. When treated with control PVA suspension, 27% of larvae died, and when treated with the blastospore PVA suspension, the mortality of larvae abruptly declined after 4 days and stopped after 6 days. We assume that in these treatments larvae and blastospores lost their vigor and virulence due to methodological and/or dose-dependent effects, as larvae and blastospores in all other (control) treatments remained viable and virulent. Moreover, the PVA suspension is not intended to be applied in the field but served as control only. Detached blastospores acted slightly slower, most likely due to the reduced cell viability caused by rapid rehydration (Minogue 1981; Kosanke et al. 1992). However, and most importantly, blastospores were not impaired but were highly lethal when applied as bead coating.

The overall virulence of *M. brunneum* against *T. molitor* was in good accordance with other studies, e.g. Oreste et al. (2012), who determined an average LT_50_ of 5.8 for four different *M. anisopliae* isolates (Oreste et al. 2012).

There is no explanation yet, why survival on the starch and AK beads differed significantly. But, more importantly, both formulations were equally lethal, indicating a dose threshold above which neither a higher level of control nor an economic benefit can be achieved. Nevertheless, this threshold applies exclusively to *T. molitor* and must be determined for relevant pests in potato cultivation, such as *Agriotes* spp. (Vernon and van Herk 2013), or other targeted arthropods.

In conclusion, the adjuvants of the PVA-based coating, PEG and Lec, highly improved the drying survival of blastospores. Moreover, the virulence of the blastospores was preserved, hence the developed blastospore coating significantly accelerated the kill effect of AK beads. To our knowledge, this is the first report on PVA-based coatings used for controlled blastospore release as a fast-acting biological pesticide. Our findings can be implemented to boost the enormous potential of regular AK formulations, coated seeds, or other biological pest control agents, as a sustainable alternative to standard chemical insecticides for integrated pest management.

Additional studies are necessary to (i) identify the optimal coating composition, (ii) transfer the formulation process to technical scale and subsequently (iii) evaluate the formulation’s efficacy under field conditions.

## Supplementary Information

**Additional file 1: Figure S1.** Blastospores and conidia survived equally in polyvinyl alcohol. Either pure PVA 4-88 4% or PVA 4-88 4%/PEG4000 2%/soy lecithin 0.8% were mixed with aerial conidia or blastospores and dried at 60 °C for 40 min in a ventilated drying cabinet to form thin films. Films were dissolved in 1 mL 0.9% NaCl and CFU were determined via standard plate counting on semi-selective PDA. Sealed Petri dishes were incubated at 23 °C for 14 days (n = 3). The extent to which conidia as benchmark and blastospores survive in thin PVA films was investigated. Survival was generally very low in pure PVA, however, PEG and lecithin increased both the conidia and blastospore survival. These findings indicate that blastospores can be used as active ingredient in a PVA coating. **Figure S2.** PVA films were dried to constant weight except for PVA 10-98. Either pure PVA 4% or PVA 4%/PEG4000 2%/soy lecithin 0.8% were dried according to standardized drying at 60 C for 20 min and at 40 °C for 35 min and subsequently re-dried at 90 °C for 60 min in a ventilated drying cabinet. The weight was determined prior to and after re-drying. Asterisks indicate significant differences according to Equivalence Test with Paired Data (n = 5–7). **Figure S3.** The remaining water content in dried PVA films tended to increase with increasing molecular weight and degree of hydrolysis. Either pure PVA 4% or PVA 4%/PEG4000 2%/soy lecithin 0.8% were dried according to standardized drying at 60 C for 20 min and at 40 °C for 35 min and subsequently re-dried at 90 °C for 60 min in a ventilated drying cabinet. The remaining water content was calculated as the weight difference before (t1) and after re-drying (t2) in relation to the weight after drying (t1) (n = 5–7). **Figure S4.** Images revealed a uniform, homogenous coating that dissolves completely within seconds. Dried uncoated calcium alginate/starch beads (a) were coated with ink-blended polyvinyl alcohol 4–88/polyethylene glycol/lecithin (b) and finally rinsed with water (c). **Table S1.** Percent mortality of *Tenebrio molitor* larvae either infected or uninfected with *M. brunneum* CB15-III, and percent survival depending on treatments. Dead individuals which exhibited no growth or one with another fungus after incubation on Potato Dextrose Agar were considered as uninfected with *M. brunneum*, including those that exhibited only very little *M. brunneum* growth. **Figure S5.** Agarose gel after PCR verified infection with *M. brunneum* CB15-III with the relevant fragment at 0.279 kilobases.

## Data Availability

The datasets generated during and/or analyzed during the current study are available from the corresponding author on reasonable request.
