# Supplementary material for: Polyvinyl alcohol coating releasing fungal blastospores improves kill effect of attract-and-kill beads
Source: AMB Express. 2023 Jul 11;13:72. doi: 10.1186/s13568-023-01575-2 (PMC10335999; doi:10.1186/s13568-023-01575-2)
Supplement: Supplementary file 1 — Additional file 1: Figure S1. Blastospores and conidia survived equally in polyvinyl alcohol. Either pure PVA 4-88 4% or PVA 4-88 4%/PEG4000 2%/soy lecithin 0.8% were mixed with aerial conidia or blastospores and dried at 60 °C for 40 min in a ventilated drying cabinet to form thin films. Films were dissolved in 1 mL 0.9% NaCl and CFU were determined via standard plate counting on semi-selective PDA. Sealed Petri dishes were incubated at 23 °C for 14 days (n = 3). The extent to which conidia as benchmark and blastospores survive in thin PVA films was investigated. Survival was generally very low in pure PVA, however, PEG and lecithin increased both the conidia and blastospore survival. These findings indicate that blastospores can be used as active ingredient in a PVA coating. Figure S2. PVA films were dried to constant weight except for PVA 10-98. Either pure PVA 4% or PVA 4%/PEG4000 2%/soy lecithin 0.8% were dried according to standardized drying at 60 C for 20 min and at 40 °C for 35 min and subsequently re-dried at 90 °C for 60 min in a ventilated drying cabinet. The weight was determined prior to and after re-drying. Asterisks indicate significant differences according to Equivalence Test with Paired Data (n = 5–7). Figure S3. The remaining water content in dried PVA films tended to increase with increasing molecular weight and degree of hydrolysis. Either pure PVA 4% or PVA 4%/PEG4000 2%/soy lecithin 0.8% were dried according to standardized drying at 60 C for 20 min and at 40 °C for 35 min and subsequently re-dried at 90 °C for 60 min in a ventilated drying cabinet. The remaining water content was calculated as the weight difference before (t1) and after re-drying (t2) in relation to the weight after drying (t1) (n = 5–7). Figure S4. Images revealed a uniform, homogenous coating that dissolves completely within seconds. Dried uncoated calcium alginate/starch beads (a) were coated with ink-blended polyvinyl alcohol 4–88/polyethylene glycol/lecithin (b) and fin [file 13568_2023_1575_MOESM1_ESM.pdf]

AMB Express

Polyvinyl alcohol coating releasing fungal blastospores improves kill effect of attract-and-kill beads

Katharina M. Hermann[1,2], Alexander Grünberger[2], Anant V. Patel[1]

[1] Faculty of Engineering and Mathematics, Fermentation and Formulation of Biologicals and Chemicals, Hochschule Bielefeld – University of Applied Sciences and Arts, Bielefeld, Germany

[2] Faculty of Technology, Multiscale Bioengineering, Bielefeld University, Bielefeld, Germany

Corresponding author:

Anant V. Patel

Hochschule Bielefeld – University of Applied Sciences and Arts, Bielefeld Institute of Applied Materials Research, Fermentation and Formulation of Biologicals and Chemicals, Interaktion 1, 33619 Bielefeld, Germany

Phone number: +49 521 106 7318

Email: anant.patel@fh-bielefeld.de

Supplementary information

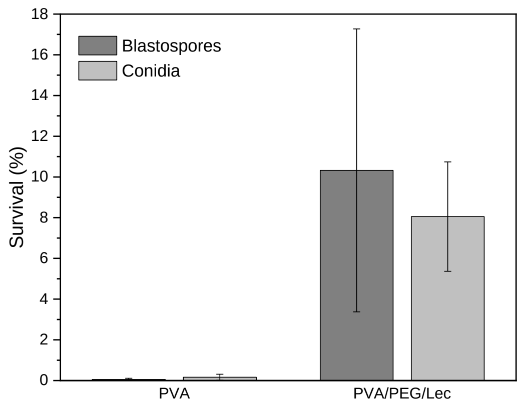

Fig. S1 Blastospores and conidia survived equally in polyvinyl alcohol. Either pure PVA 4-88 4 % or PVA 4-88 4 %/PEG4000 2 %/soy lecithin 0.8 % were mixed with aerial conidia or blastospores and dried at 60 °C for 40 min in a ventilated drying cabinet to form thin films. Films were dissolved in 1 mL 0.9 % NaCl and CFU were determined via standard plate counting on semi-selective PDA. Sealed Petri dishes were incubated at 23 °C for 14 days (n=3). The extent to which conidia as benchmark and blastospores survive in thin PVA films was investigated. Survival was generally very low in pure PVA, however, PEG and lecithin increased both the conidia and blastospore survival. These findings indicate that blastospores can be used as active ingredient in a PVA coating.

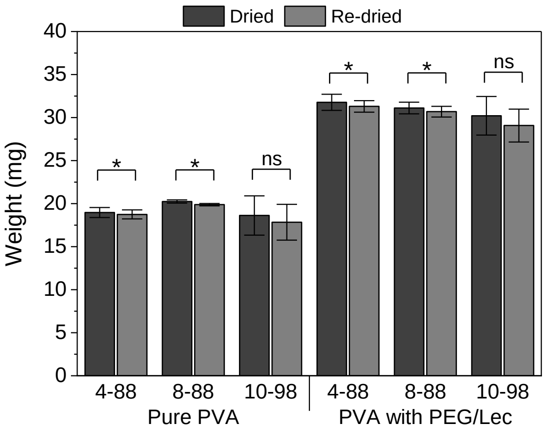

Fig. S2 PVA films were dried to constant weight except for PVA 10-98. Either pure PVA 4 % or PVA 4 %/PEG4000 2 %/soy lecithin 0.8 % were dried according to standardized drying at 60 C for 20 min and at 40 °C for 35 min and subsequently re-dried at 90 °C for 60 min in a ventilated drying cabinet. The weight was determined prior to and after re-drying. Asterisks indicate significant differences according to Equivalence Test with Paired Data (n=5–7).

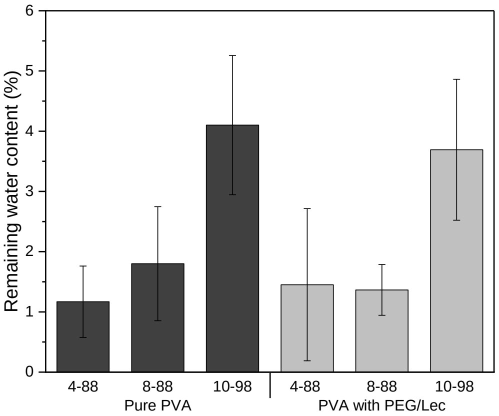

Fig. S3 The remaining water content in dried PVA films tended to increase with increasing molecular weight and degree of hydrolysis. Either pure PVA 4 % or PVA 4 %/PEG4000 2 %/soy lecithin 0.8 % were dried according to standardized drying at 60 C for 20 min and at 40 °C for 35 min and subsequently re-dried at 90 °C for 60 min in a ventilated drying cabinet. The remaining water content was calculated as the weight difference before (t1) and after re-drying (t2) in relation to the weight after drying (t1) (n=5–7).

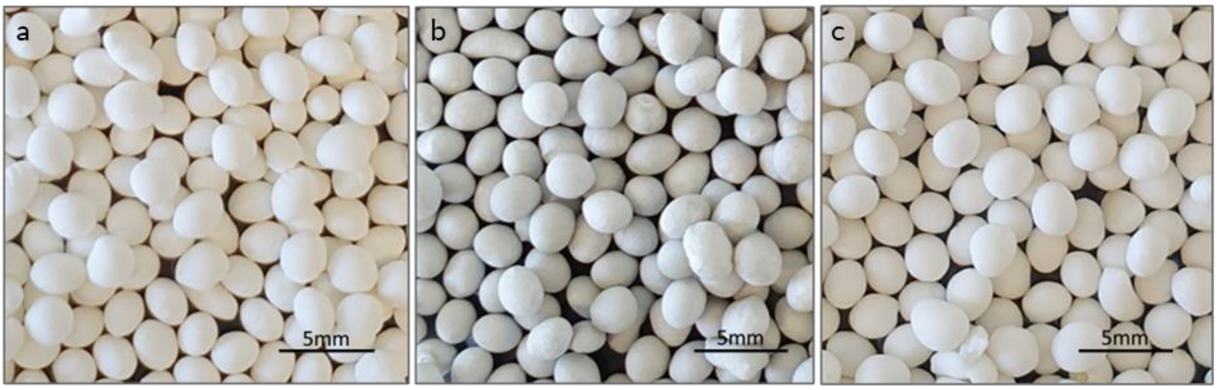

Fig. S4 Images revealed a uniform, homogenous coating that dissolves completely within seconds. Dried uncoated calcium alginate/starch beads (a) were coated with ink-blended polyvinyl alcohol 4-88/polyethylene glycol/lecithin (b) and finally rinsed with water (c).

Table S1 Percent mortality of *Tenebrio molitor* larvae either infected or uninfected with *M. brunneum* CB15-III, and percent survival depending on treatments. Dead individuals which exhibited no growth or one with another fungus after incubation on Potato Dextrose Agar were considered as uninfected with *M. brunneum*, including those that exhibited only very little *M. brunneum* growth.

Treatment	Dead and infected (%)	Dead but uninfected (%)	Survival (%)
**Suspensions**			
Control (neg. ctrl)	0	10	90
BS NaCl (pos. ctrl)	100	0	0
Control PVA	5	27	68
BS PVA	43	7	50
BS detached	95	5	0
**Bead formulations**			
Starch	10	10	80
Starch Coat	0	0	100
Starch BS	95	5	0
AK	100	0	0
AK BS	100	0	0

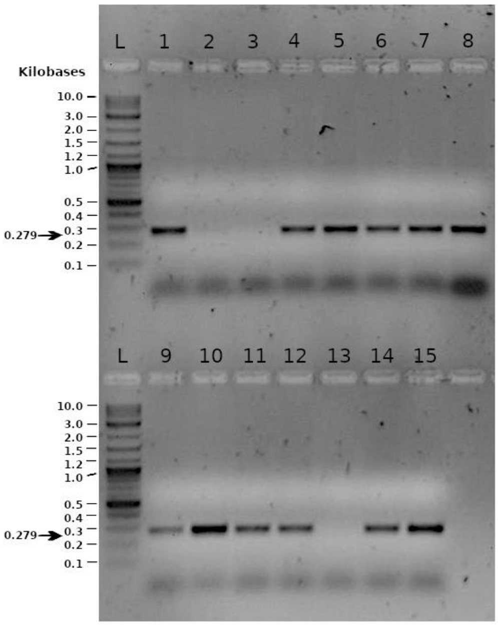

Fig. S5 Agarose gel after PCR verified infection with *M. brunneum* CB15-III with the relevant fragment at 0.279 kilobases. No. 2 and 3 revealed contamination of the corresponding sample with unknown fungi, and no. 4 and no. 9 revealed contamination of the corresponding sample with *M. brunneum* CB15-III. Random samples and those with abnormal fungal morphology and unexpected fungal growth were investigated. For PCR, DNA was extracted with a DNA extraction kit (DNeasy Plant Pro, Qiagen GmbH, Hilden, Germany). According to instructions, 20-50 mg moist biomass, either mycelium and/or conidia, was disrupted with a steel ball lysing matrix tube in lysis buffer in a ball mill at 24 Hz for 2 min, twice. Extracted DNA was eluted in 50 µL elution buffer and stored at -20 °C until further use. Subsequent qualitative PCR was performed with a ready-to-use master mix (TopTaq Master Mix Kit, Qiagen GmbH, Hilden, Germany). PCR reactions contained 3 µL DNA, 10 µL TopTaq Mastermix 10x, 2 µL CoralLoad, 1 µL of each primer and 3 µL RNase-free water. The PCR cycling parameters included an initial denaturation for 2 min at 95 °C, followed by 30 cycles of 30 s at 95 °C, 30 s at 62 °C and 40 s at 68 °C, concluded with 5 min incubation at 68 °C (TProfessional Basis, Biometra Analytik Jena GmbH, Jena, Germany). PCR results were assessed by gel electrophoresis at 60 V in a 2 % agarose gel stained with ROTI® GelStain. L: Ladder (Quick-Load® Purple 1 kb Plus DNA). 1: BS NaCl, 2–4: PVA Control, 5–7: S BS, 8: AK BS, 9: S, 10: BS detached, 11: *M. brunneum* CB15-III conidia from agar plate as positive control, 12: *M. brunneum* CB15-III conidia from mealworm, 13: Negative control, 14: Control DNA extraction, 15: Control PCR Primer.
